# Phenotypic and genotypic characterization of antibiotic-resistant in *Escherichia coli* isolates from patients with diarrhea

**Published:** 2019-06

**Authors:** Mojtaba Bonyadian, Sara Barati, Mohammad Reza Mahzounieh

**Affiliations:** 1 Department of Health and Food Quality Control, Institute of Zoonoses Research, Shahrekord University, Shahrekord, Iran; 2 Department of Microbiology, Ahvaz Branch, Islamic Azad, University, Ahvaz, Iran; 3 Department of Pathobiology, Institute of Zoonoses Research, Shahrekord University, Shahrekord, Iran

**Keywords:** Antibiotics resistance, Phenotypic, Genotypic, *Escherichia coli*, Diarrhea

## Abstract

**Background and Objectives:**

*Escherichia coli* is a common enteric pathogen of human and livevestock. Antibiotic resistance is the main concern of public health. The aim of this study was to detect this bacterium in stool samples of diarrheal patients and identify the phenotypic and genotypic characterizations of antibiotic-resistant isolates such as *dfrA1, sul1, citm, tetA, qnr, aac(3)-IV* in Shahrekord.

**Materials and Methods:**

Two hundred fifty diarrheal stool samples from patients were collected. Microbiological and biochemical examinations were done to detect *E. coli*. Phenotypic and genotypic antibiotic resistance of the isolates were determined using dick diffusion method and polymerase chain reaction (PCR), respectively.

**Results:**

Among 114 *E. coli* isolates, the least resistance was for gentamicin (0%) and the most resistance was for trimethoprim (79.8%). The resistance to sulfamethoxazole, ciprofloxacin, ampicillin, and tetracycline were 71.05%, 10.5%, 52.63%, and 3.5% respectively. The results of PCR assay revealed that 10 isolates contain *sul1*, 49 isolates harbor *citm*, 8 isolates *tetA*, 36 isolates *dfrA1*, 11 isolates *qnr* genes but there was no isolate with *aac(3)-IV* gene. In comparison between phenotypic and genotypic of the isolates revealed that *citm, tetA, dfrA1, qnr, sul1, aac(3)-IV* genes covered 42.98%, 7.01%, 31.57%, 9.64%, 8.7%, 0% of the antibiotic resistance, respectively.

**Conclusion:**

Our results revealed that all isolates harbor one or more antibiotic resistance genes and that the PCR is a fast practical and appropriate method to determine the presence of antibiotic resistance genes.

## INTRODUCTION

There is worldwide concern about the appearance and rise of bacterial resistance to commonly used antibiotics. In this regard program for monitoring, resistance has been implemented in many countries (1, 2, 3). It has been demonstrated that diarrhoeagenic *E. coli* strains are categorized into specific groups based on virulence properties, mechanisms of pathogenicity, clinical syndromes, and distinct O: H serotypes (4). A number of *E. coli* strains are recognized as important pathogens of colibacillosis in poultry and some of them can cause severe human diseases such as hemorrhagic colitis and hemolytic uremic syndrome (5–7). The six main categories include enteropathogenic *E. coli* (EPEC), enterotoxigenic *E. coli* (ETEC), enteroinvasive *E. coli* (EIEC), enteroaggregative *E. coli* (EAEC), enterohemorrhagic *E. coli* (EHEC or STEC), diffuse adhering *E. coli* (DAEC) (8). Enterotoxigenic *E. coli* is a common cause of diarrheal disease in developing countries. The enteric pathogens are often resistant to multiple antibiotics.

However, a large number of outbreaks of enterotoxins have also been associated with the consumption of contaminated drinking water or contact with recreational water (5–7, 9).

*E. coli* infections as a cause of disease have shown a marked increase in many countries.

*E. coli* use as an index for determining fecal contamination in water and foods. Foods contaminated with antibiotic-resistant bacteria could be a major threat to public health as there is possibility that genes encoding antibiotic resistance determinants that are carried on mobile genetic elements may be transferred to other bacteria of human clinical significance. *E. coli* is a candidate vehicle for such transfers because of its diversity and also because it survives as common flora in the gastrointestinal tracts of both humans and animals. They are sensitive to selection pressure exerted by antibiotic usage and carry genetic mobile elements to achieve such transmission (10). In addition, the lack of stringent controls on antimicrobial use in human health and particularly in animal production systems increase the risk of foodborne microbes harboring an array of resistance genes. In many countries for the purpose of protecting the health of humans as well as animals, treatment of illnesses caused by this bacterium often requires antimicrobial therapy (1, 2, 3). The decision to use antimicrobial therapy depends on the susceptibility of the microorganisms and the pharmacokinetics of the drug for achieving the desired therapeutic concentration at the site of infection and thus clinical efficacy (11).

This study was conducted to baseline profile of antimicrobial resistance of *E. coli* isolated from patients with diarrhea. We undertook this study to identify the presence of isolates of *E. coli* from stool samples from patients with diarrhea in Shahrekord and to characterize the genes and comparison between the phenotypic and genotypic characterization of antibiotic-resistant strains.

## MATERIALS AND METHODS

### Sample collection.

A total of 250 diarrheal fecal samples from the patients were collected in Hajar hospital of Shahrekord. The questionnaire was prepared and filled by patients.

### Isolation of *E. coli*.

MacConky agar and Salmonella Shigella agar (Merck, Germany), were used to detect *E. coli*. A swab of fecal sample was cultured on MCA and SS agar and incubated for 24 h at 37°C. Complete biochemical identification (Gram staining, oxidase, indole, Simon’s citrate and urease) was used to identify the isolated organism. Bacteriological examinations were done on non-lactose fermenting colonies to confirm the major causes of diarrhea e.g. *Salmonellae* and *Shigella* (12, 13).

### Antimicrobial susceptibility.

Antimicrobial susceptibility testing was carried out by the disk diffusion method according to the Clinical and Laboratory Standard Institute guidelines (CLSI, 2018) (14, 15).

### Detection of *citm, tetA, dfrA1, qnr, sul1, aac (3)-IV* genes.

Total DNA of the isolates was extracted using the Genomic DNA purification kit (Fermentas, Germany). The isolated DNA was suspended in 50 ul of Tris-EDTA (TE) buffer at pH 8. Two microlitres of eluting were used as DNA template in PCR assay. PCR was performed using 6 primer sets (Cinagen, Iran) that detect antibiotic resistance genes.

The set of primers used for each gene is shown in Table 1.

**Table 1.** Primers sequences used in PCR and expected sizes of products

	**Target**	**Sequence (5-3)**	**Size bp**	**Annealing Temp**
	*Aac(3)-IV-F*	CTT CAG GAT GGC AAG TTG GT	286	54
	*Aac(3)-IV-R*	TCA TCT CGT TCT CCG CTC AT	286	
	*Sul1-F*	TTC GGC ATT CTG AAT CTC AC	822	47
	*Sul1-R*	ATG ATC TAA CCC TCG GTC TC	822	
	*CITM-F*	TGG CCA GAA CTG ACA GGC AAA	462	49
	*CITM-R*	TTT CTC CTG AAC GTG GCT GGC	462	
	*TetA–F*	GGT TCA CTC GAA CGA CGT CA	577	55
	*TetA-R*	CTG TCC GAC AAG TTG CAT GA	577	
	*dfrA1-F*	GGA GTG CCA AAG GTG AAC AGC	367	49
	*dfrA1-R*	GAG GCG AAG TCT TGG GTA AAA AC	367	
	*Qnr-F*	GGG TAT GGA TAT TAT TGA TAA AG	670	55
	*Qnr-R*	CTA ATC CGG CAG CAC TAT TTA	670	

The presence of genes associated with resistance to ampicillin (*citm*), tetracycline (*tetA*) trimethoprim (*dfrA1*), quinolones (*qnr*), gentamicin (*aac(3)-I* V) and sulfonamides (*sul1*) was determined by PCR. Reactions were performed in a total volume of 25 μl, including 1.5 ml MgCl_2_, 50 mM KCl, 10 mM Tris-HCl (pH 9.0), 0.1% Triton X-100, 1 μl of each dNTP, 1 μl primers, 0/2 IU of Taq DNA polymerase (Fermentas), and 2 μl of DNA. Amplification reactions were carried out using a DNA thermo-cycler (Bio-Rad) as follows: Five min at 95°C, 35 cycles each consisting of 1 min at 94°C, 30 s at ∼55°C and 1 min at 72°C, followed by a final extension step of 5 min at 72°C. Amplified samples were analyzed by electrophoresis in 1.5% agarose gel and stained by ethidium bromide. A molecular weight marker with 100 bp increments (100 bp DNA ladder, Fermentas) was used as a size standard (10, 16).

## RESULTS

One hundred fourteen (34.2%) lactose fermenting and pink colonies were isolated on MCA and were confirmed as *E. coli* by biochemical and microbiological tests. Also, the antibiotic resistance of the isolates was evaluated. In phenotype, the least resistance was for gentamicin (0%) and the most resistance was for trimethoprim (79.8%). The resistance to cotrimoxazole, ciprofloxacin, ampicillin, and tetracycline was found in 71.05%, 10.5%, 52.63%, 3.5% of isolates respectively (Table 2).

**Table 2.** Percentage of antibiotic resistance of *E. coli* isolated from diarrheal samples

**Antibiotics**	**(S)**	**(I)**	**(R)**
Tetracyclin	31.5	64.9	3.5
Gentamicin	89.4	10.5	0
Ampicillin	12.28	35.08	52.63
Ciprofloxacin	75.4	14.03	10.5
Sulphametoxazole	7.01	21.92	71.05
Trimethoprim	19.29	0.87	79.8

S: Susceptible, I: Intermediate, R: Resistance

In PCR assay, 10 isolates contain *sul1*, 49 isolates contain *citm*, 8 isolates contain *tetA*, 36 isolates contain *dfrA1*, 11 isolates contain *qnr* genes but there was no isolate with *aac(3)-IV* gene (Table 3).

**Table 3.** Antibiotic resistance genes in *E. coli* isolated from diarrheal samples

***qnr***	***dfrA1***	***tetA***	***citM***	***sul1***	***aac(3)-IV***
11 (9.64%)	36 (31.57%)	8 (%7.01)	49 (42.98%)	10 (8.7%)	0 (0%)

Also, the results showed that the single resistance found in 27.19%, double resistance in 18.42%, triple resistance in 6.14% and quadruple resistance in 4.38% of the isolates (Table 4).

**Table 4.** Presence of multiple resistance genes in isolated *E. coli*

**Number**	**Gene**		**Type of resistance**
9	*dfrA1*	31 (%27.19)	Single
17	*citm*		
5	*qnr*		
16	*dfrA1-Citm*	21 (18.42%)	Double
1	*dfrA1-Sul1*		
2	*Sul1-Citm*		
2	*qnr-citm*		
2	*dfrA1-Sul1-citm*	7 (6.14%)	Triple
3	*dfrA1-tet-citm*		
1	*Sul1-citm-tet*		
1	*Sul1-Citm-Qnr*		
2	*dfrA1-sul1-citm-tet*	5 (4.38%)	Quadruple
1	*dfrA1-sul1-citm-qnr*		
2	*dfrA1-citm-tet-qnr*		

Comparison between phenotypic and genotypic of the isolates revealed that *citm, tetA, dfrA1, qnr, sul1, aac(3)-IV* genes covered 42.98%, 7.01%, 31.57%, 9.64%, 8.7%, 0% of the antibiotic resistance in the isolates, respectively.

## DISCUSSION

Our findings showed that *E. coli* were the major cause of human enteric infections in this area of Iran. A lot of researches have been run in recent years on detection, identification, and molecular characterization of antibiotic resistance genes, which has led to a more accurate assessment of the role of these bacteria in human disease outbreaks and the transmission of infection from an animal reservoir. Most information on risk factors associated especially *E. coli* infection has come from outbreak investigations. Among identified dietary risk factors, foods of bovine origin, particularly undercooked ground beef, have been a frequently implicated source. Non-dietary risk factors including person-to-person transmission in day-care settings or swimming in contaminated water have also been documented (17, 18). In our study, about half of the isolated *E. coli* belonged to *citm* gene, and more than of 40% of the isolates were positive for the *dfrA* gene, but none of them carried *aac(3)-IV* gene. Also, more than 10% of the isolates were positive for *sul1, tetA, qnr* genes. Lien et al. (2018) reported that resistance to at least one antibiotic was detected in 83% of *E. coli* isolated from hospital wastewater in Vietnam; multidrug resistance was found in 32% of the isolates. The highest resistance prevalence was found for co-trimoxazole (70%) and the lowest for imipenem (1%), (19). Hassan (2018) revealed that all the *E. coli* strains isolated from patients and food were highly resistance to penicillin, amoxicillin-clavulanic and erythromycin with a percentage of 100%, while the resistance to gentamicin, ampicillin, oxytetracycline, chloramphenicol, norfloxacin, trimethoprim, and nalidixic acid were 83%, 75%, 65.3%, 55.8%, 36.5%, 30.7% and 26.9% respectively (20). Weiss et al. (2018) reported that 29.6% of the isolated *E. coli* were resistant to at least one of 11 antibiotics tested. The frequency of resistance reached 20.3% of isolates for trimethoprim-sulfamethoxazole but was nearly zero for the less commonly available antibiotics ciprofloxacin (0.4%), gentamicin (0.2%), and ceftiofur (0.1%). The frequency of resistance was 57.4% in isolates from people, 19.5% in isolates from domestic animals, and 16.3% in isolates from wild nonhuman primates (21).

A study on *E. coli* isolated from fecal samples of children in Taiwan (2018) showed that the rates of resistance to ampicillin, amoxicillin + clavulanate, trimethoprim-sulfamethoxazole, and cefazolin were 70, 65.6, 47.1 and 32.5%, respectively (22).

Shehata, et al. (2017) indicated that all the isolated *E. coli* from fecal samples of human and chicken in Egypt show high resistance to multiple antibiotics. Strains of *E. coli* from human were highly resistant to ampicillin (72.7%). The antibiotic resistance genes *bla*_OXA_ *, shv, dhfrV, dhfrI, cmlA* and *cat1* were detected in both human isolates and animal isolates (23).

Our study revealed that the presence of *E. coli* in diarrheal stool samples of patients emphasizing the need of using protocol for detection of all serotypes of *E. coli* from human, animals and meat products in clinical and food microbiology laboratories. The mechanism of spread of antibiotic resistance from food animals to humans remains controversial.

However, veterinary practitioner has a limited choice of antimicrobials for use in the poultry industry due to antimicrobial resistance issues and human health concerns.

Moreover, the repeated and unsuitable use of antibiotics has led to an increasing rate of antimicrobial resistance (16). Now different PCR protocols for detection of *E. coli* are available making a diagnosis of *E. coli* infections possible.

In conclusion, our results showed that antibiotic-resistant *E. coli* was the main bacterial pathogen causing diarrhea in this part of Iran and advanced detection methods like PCR need to be used in microbiology to confirm the antibiotic resistance genes as well as disk diffusion method.
